# Making heads or tails of mitochondrial membranes in longevity and aging: a role for comparative studies

**DOI:** 10.1186/2046-2395-3-3

**Published:** 2014-03-03

**Authors:** Teresa G Valencak, Vian Azzu

**Affiliations:** 1 Research Institute of Wildlife Ecology, Veterinary University Vienna, Savoyenstrasse 1, 1160 Vienna, Austria; 2 Institute of Genetics and Developmental Biology, State Key Laboratory of Molecular Developmental Biology, Chinese Academy of Sciences, Beichen Xi Lu, Chaoyang, Beijing, China; 3 Christ’s College & Department of Medicine, University of Cambridge, Cambridge CB2 3BU, UK

**Keywords:** Mitochondria, comparative biology, endotherm, ectotherm, fatty acid membrane composition, longevity, maximum lifespan

## Abstract

Mitochondria play vital roles in metabolic energy transduction, intermediate molecule metabolism, metal ion homeostasis, programmed cell death and regulation of the production of reactive oxygen species. As a result of their broad range of functions, mitochondria have been strongly implicated in aging and longevity. Numerous studies show that aging and decreased lifespan are also associated with high reactive oxygen species production by mitochondria, increased mitochondrial DNA and protein damage, and with changes in the fatty acid composition of mitochondrial membranes. It is possible that the extent of fatty acid unsaturation of the mitochondrial membrane determines susceptibility to lipid oxidative damage and downstream protein and genome toxicity, thereby acting as a determinant of aging and lifespan. Reviewing the vast number of comparative studies on mitochondrial membrane composition, metabolism and lifespan reveals some evidence that lipid unsaturation ratios may correlate with lifespan. However, we caution against simply relating these two traits. They may be correlative but have no functional relation. We discuss an important methodology for body mass and phylogenetic correction in comparative studies.

## Review

### Introduction

#### A brief history of longevity hypotheses

Over a century ago, Max Rubner observed for six animal species that larger animals had a slower metabolic rate per unit mass and a longer lifespan compared with smaller animals. Rubner [1] Later work by Kleiber [2] and others in the 1930s supported this finding for a larger range of species. This led to several hypotheses suggesting that aging and longevity are processes that are regulated by metabolic rate.

Raymond Pearl suggested that animal tissues had a finite number of chemical reactions available, which on exhaustion led to mortality [3]. Therefore, organisms with a higher metabolism per unit mass would age and die sooner. This became known as the ‘rate of living hypothesis’.

As scientists were just beginning to understand free radical biology in the 1950s [4], Denham Harman suggested a mechanism linking metabolic rate to aging and lifespan [5]. He proposed that reactive oxygen species, being the products of metabolism, would cause cumulative damage and result in aging followed by death. This ‘free radical hypothesis of aging’ actually echoed suggestions made earlier in the century by Elie Metchnikoff that ‘senility’ may be a consequence of ‘waste’ products of metabolism [6].

Studies showing that metabolic rate-matched [7] or size-matched animals had different lifespans [7,8] undermined the rate of living hypothesis and suggested that metabolic rate is not the exclusive determinant of lifespan. However, these early observations contributed to the question of why metabolic rate varies substantially across species, especially between size-matched endotherms (higher metabolic rate) and ectotherms (lower metabolic rate) [7]. Brand and colleagues examined metabolic rate differences in hepatocytes isolated from a mammal (a rat) and a reptile (a lizard) [9], and found that the respiration rate was fivefold higher in rat hepatocytes, possibly due to an increased amount of n-3 polyunsaturated fatty acid (PUFA) in the mitochondrial membranes [9]. However, they noted no difference in the percentage of respiration rate dedicated to processes such ATP production, proton leak across the mitochondrial inner membrane and maintenance of Na/K antiporter activity at the plasma membrane [9]. The variation in amplitude but not distribution of metabolic rate across species and its correlation with mitochondrial phospholipid composition [10], led Hulbert and Else to propose that membrane composition acts as a ‘pacemaker for metabolism’ [11]. Specifically, they postulated that membrane polyunsaturation, higher in the tissues of mammals in comparison with reptiles, would increase the molecular activity of membrane proteins thereby increasing cellular metabolic activity. Although this hypothesis held true between some species, it did not when birds were introduced into the equation, as birds have an increased metabolic rate compared to mammals, but lower membrane polyunsaturation [12].

Because of the broad but not perfect correlations of membrane fatty acid levels with metabolism, and metabolism with lifespan, a natural line of investigation developed looking at membrane composition with respect to lifespan, thus developing into the ‘homeoviscous-longevity adaptation’ [13] and later, the ‘membrane pacemaker hypothesis of aging’ [14]. These hypotheses linked membrane fatty acid unsaturation to susceptibility to oxidative damage, the propagation of which is associated with aging and mortality. In light of an increasing number of studies that support and conflict with these hypotheses, our review seeks to explore the evidence for the link between mitochondrial phospholipid and fatty acid composition, metabolism and lifespan. We discuss the roles for allometric (body size) and phylogenetic (species relatedness) corrections when making comparisons between different species [15,16].

### Membrane landscapes in mitochondria

Mitochondria are intracellular organelles whose primary function is metabolic energy transduction and ATP synthesis. They also play vital roles in intermediate molecule metabolism, metal ion (calcium and iron) homeostasis, programmed cell death and regulation of the production of reactive oxygen species (ROS) [17]. As a result of their broad range of functions, mitochondria have been strongly implicated in aging and longevity (reviewed in [18]). In fact, numerous studies have shown that longevity or lifespan may be affected by mitochondrial ROS production [5], mitochondrial DNA damage [19] and mitochondrial membrane fatty acid composition [11], the latter will be the focus of this article.

#### Background to phospholipids and fatty acids

**Figure 1:**
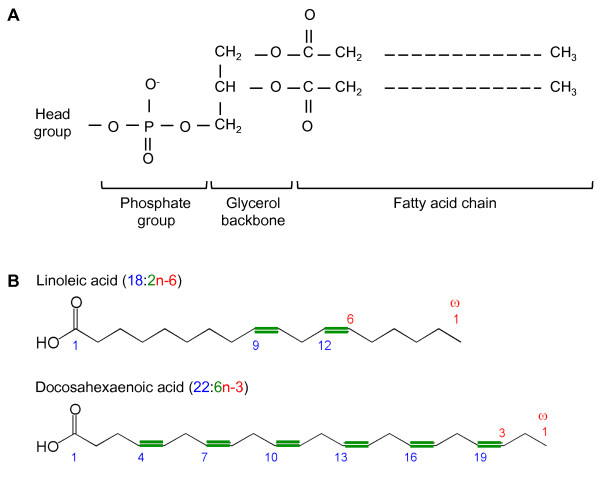
**Glycerophospholipids and fatty acids in mitochondrial membranes. (A)** Phospholipid molecules are composed of a glycerol backbone to which are attached (sn-1 and sn-2 hydroxyl groups) fatty acid chains of variable length and number of double bonds. A hydrophilic head group can be attached via a phosphodiester bond formed at the sn-3 position. **(B)** The first carbon in carboxylic acids is at the alpha end. The last carbon is at the omega (n) end. For omega-3 (n-3) fatty acids, such as docosahexaenoic acid, the first double bond is three carbons from the omega end. The first double bond for omega-6 (n-6) fatty acids, such as linoleic acid, is at the sixth carbon from the omega end. Examples of nomenclature are shown: the carbon chain length beginning from the alpha end is shown in blue. The carbon double bonds are shown in green. The position of the first carbon double bond from the omega end is shown in red.

Membrane lipids can broadly be classified as glycerophospholipids, sphingolipids or sterols. These lipid moieties may be complexed to sugars and proteins in a cell membrane. The vast majority of mitochondrial membranes are composed of glycerophospholipids [20], which contain a glycerol backbone, a hydrophilic head group and fatty acid chains (Figure 1A). Naturally occurring fatty acids typically contain 4 to 28 aliphatic carbons of variable length and saturation: saturates contain no carbon double bonds, monounsaturates contain one double bond and polyunsaturates more than one. Figure 1B illustrates fatty acid structure and nomenclature.

Extensive work by Daum and colleagues [21,22] has shown that the mitochondrial inner membrane is composed of all the major classes of membrane phospholipids, including phosphatidylcholine, phosphatidylethanolamine, phosphatidylinositol, phosphatidylserine, phosphatidic acid, phosphatidylglycerol and cardiolipin (CL) [22] (Table 1). Mitochondria contain a few other membrane lipids such as sphingolipids and sterols [23], the notable exception being mitochondria involved in steroid synthesis [24].

**Table 1:** **Lipid composition of mitochondrial outer (MOM) and inner (MIM) membranes in mammals, plants and yeast**^**a**^

	**Mammalian cells**		**Plant cells**		**Yeast**	
	**(Rat liver)**		**(Cauliflower)**		**( *Saccharomyces cerevisiae *)**	
	**MOM**	**MIM**	**MOM**	**MIM**	**MOM**	**MIM**
Phospholipid (mg/mg protein)	0.45	0.2	0.63	0.41	0.91	0.15
	Percentage of phospholipid					
Phosphatidylcholine	54	40	47	42	46	38
Phosphatidylethanolamine	29	34	27	38	33	24
Phosphatidylinositol	13	5	23	5	10	16
Phosphatidylserine	2	3	-	-	1	4
Cardiolipin	<1	18	3	15	6	16
Phosphatidic acid	1	-	-	-	4	2

^a^Mitochondrial phospholipid levels were measured relative to mitochondrial protein levels and the individual phospholipid classes were calculated as a percentage of the total. Data from [22].

**Figure 2:**
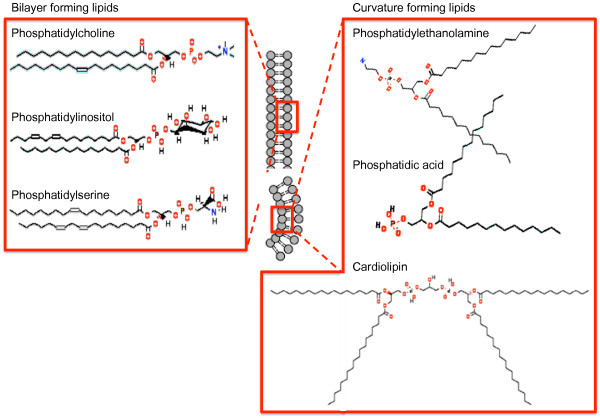
**Phospholipid structure and contribution to membrane ultrastructure.** The mixing of the head group with the fatty acid chain confers properties such as a conical shape for curvature-inducing lipids or a condensed cylindrical form for planar bilayer lipids.

The different classes of phospholipids and fatty acids confer different properties on the membrane including its ultrastructure. As shown in Figure 2, when the diameters of the hydrophilic head groups and fatty acid chains are similar, the phospholipid molecules take on a cylindrical shape that renders the molecule suitable for forming lipid bilayers. However, small hydrophilic head groups combined with large hydrophobic fatty acid chain diameters lead to a conical shape. This favours a negative curvature, which *in vitro* forms hexagonal phase structures, but which *in vivo* is likely to store curvature stress resulting in packing defects and differential lateral pressure profiles, which may affect protein function (reviewed in [25]). Curvature stress energy can affect the binding of membrane proteins within the lipid bilayer or supply energy for protein conformational changes [25]. This is particularly important for the mitochondrial phospholipid CL, whose role in metabolism and lifespan is reviewed later.

Membrane phospholipid and fatty acid compositions are influenced by diet, which can alter membrane composition by several per cent [26]. However, there are much larger species- and tissue-specific differences in fatty acid composition, suggesting an overriding and larger effect of gene expression [15]. Indeed, the fatty acid composition of mitochondrial phospholipids varies widely across species [20] and correlates with body size, basal metabolic rate and longevity [27-29].

### Does mitochondrial membrane composition affect metabolic rate and longevity?

An allometric comparison of metabolism between ectotherms and endotherms indicates that longer-lived slower metabolizing ectotherms such as lizards also have very low levels of membrane polyunsaturated fatty acids. By contrast shorter-lived endotherms, such as mice and rats, with higher metabolic rates, have highly unsaturated membranes [11]. This observation forms the basis for the ‘membrane pacemaker hypothesis of metabolism’, which posits various hypotheses to explain how membrane fatty acid composition may causally affect basal metabolic rate, and by extension longevity. It may do so by altering the function of embedded proteins [30] or by changing permeability and/or the proton leak across the inner mitochondrial membrane [10].

Several studies have shown that changes in membrane fatty acid composition affect protein function in mitochondria, for proteins such as succinate dehydrogenase [31] and cytochrome c oxidase [32], as well as other membrane proteins such as the Na/K antiporter [30], which accounts for 10% to 60% of the resting metabolic rate according to tissue type [33,34]. In particular, Wu, Else and Hulbert conducted some elegant endotherm/ectotherm crossover studies [30] to show that membrane composition and fatty acid packing in monolayers affects Na/K antiporter activity [35] and thereby metabolic rate. Whilst these studies may explain why membrane composition is linked to basal metabolism, they do not support the view that altering the function of embedded membrane proteins might affect lifespan and aging, and we are not aware of any other studies showing such a correlation. Furthermore, we suggest that empirical comparisons of membrane parameters for phylogenetically distant groups such as ectotherms and endotherms are complicated by differences in temperature regulation and weight-specific metabolism, which should be corrected for where possible.

#### Mitochondrial membrane composition affects proton leak and metabolic rate but is not associated with longevity

**Figure 3:**
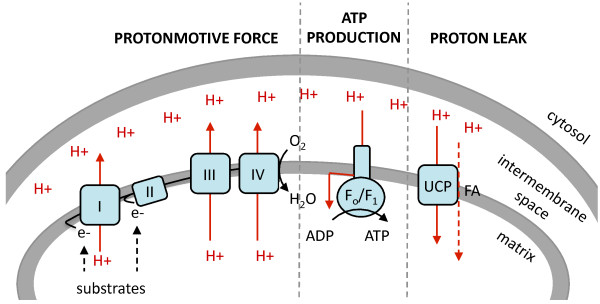
**Supply, demand and leak pathways of the protonmotive force in mitochondria.** Redox reactions at the respiratory complexes (I to IV) create a proton electrochemical gradient, which may be consumed by the F_o_/F_1_ ATPase to produce ATP or by proton leak pathways, which release energy in the form of heat. Proton leak pathways can occur through gradient-dependent cycling for example by fatty acids (FAs) or by activation of uncoupling protein (UCP). FA, fatty acid, UCP, uncoupling protein.

Figure 3 illustrates the coupling between substrate oxidation and ejection of protons by the electron transport chain from the matrix side to the intermembrane space, thus generating a protonmotive force [36]. This electrochemical gradient can then be used to drive energy (ATP) production through ATP synthase [37]. However, electrochemical transduction is not perfectly coupled [38] and protons can leak back from the intermembrane space to the matrix via various processes including passive gradient-dependent cycling carried out by membrane fatty acids or directly by activation of proteins such as the mitochondrial uncoupling proteins (UCPs) [39]. This is termed proton leak, or uncoupling.

Brookes and colleagues [40] have shown that in simplified liposome systems from the phospholipids of eight vertebrates, representing a tenfold range of mitochondrial proton leak and a threefold difference in membrane unsaturation, the mitochondrial proton leak was similar. In a subsequent study on isolated mitochondria [10], they showed that proton leak (per milligram of mitochondrial protein) correlated with increased membrane unsaturation. Conversely, a low proton leak was associated with decreased metabolism and increased monounsaturates in the membrane. Thus, Brookes *et al.* concluded that mitochondrial fatty acid composition might affect the behaviour of one or more mitochondrial inner membrane proteins and thereby might affect the proton leak [10]. Furthermore, the proton leak via the lipid portion of the mitochondrial inner membrane was estimated to be only 5% of the total membrane proton leak, again suggesting that fatty acid composition might influence the proton leak via proteins, but was not the primary mediator of the process [41]. There is now good evidence that both fatty acids, especially polyunsaturates [42], and lipid peroxidation products [43] activate uncoupling proteins. The activation of uncoupling proteins by products of reactive oxygen species is thought to act as a negative feedback loop to decrease production of such species [44]. By consuming and lowering the protonmotive force, uncoupling decreases the steady-state concentration of carriers that are likely to donate an electron to oxygen to generate ROS [39,43].

An exceptional finding to the membrane pacemaker hypothesis of metabolism is that of birds, which have a higher metabolic rate and generally live longer than size-matched mammals. One might suppose that this can be explained through mild uncoupling in birds, which would increase metabolic rate, but decrease ROS production, thus potentially explaining their longevity. However, studies have shown conflicting results in proton leak rates [10] or ROS production [8,16,45] in birds compared with size-matched mammals. The question of whether membrane lipids are directly correlative with uncoupling in mediating lifespan extension remains unanswered. Combining studies looking at membrane composition and uncoupling [46,47], and membrane composition and lifespan [14,38] requires unsafe assumptions that result in conflicting outcomes. The topic of uncoupling and lifespan is extensively reviewed elsewhere [48].

### Membrane unsaturation and peroxidation

Studies by the Pamplona and Barja group first suggested that low fatty acid unsaturation in mitochondria protects against lipid peroxidation in liver mitochondria for the long-lived pigeon compared to the shorter-lived but phylogenetically very distant rat [49]. In a subsequent study, they showed that the extent of membrane unsaturation was directly correlated with increased lifespan in several mammals [50], although their data were not specific to mitochondrial membrane composition.

**Figure 4:**
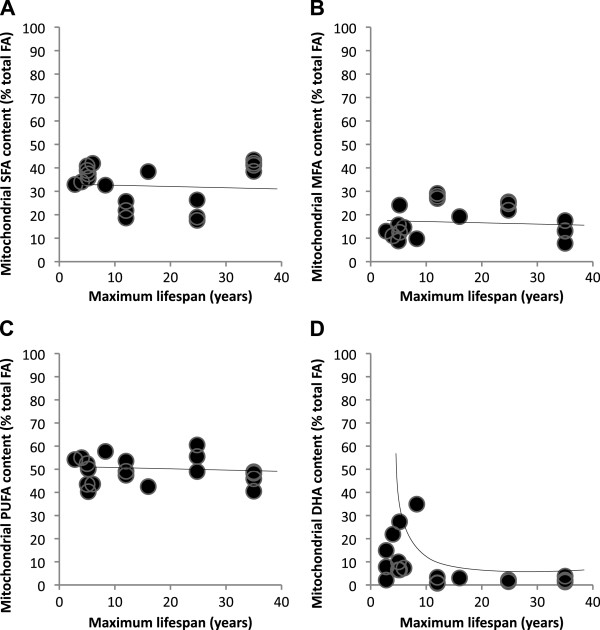
**Fatty acid contents for a range of tissues from endothermic and exothermic species. (A)** Mitochondrial saturated fatty acids. **(B)** Monounsaturated fatty acid. **(C)** Polyunsaturated fatty acids. **(D)** Docosahexaenoic acid. The data are graphical displays of the fatty acid content detailed in Table 2. These data have not undergone statistical analysis, as described in the main text. DHA, docosahexaenoic acid; FA, fatty acid; MUFA, monounsaturated fatty acid; PUFA, polyunsaturated fatty acid; SFA, saturated fatty acid.

In Table 2, we have compiled the mean mitochondria-specific content of saturated (SFA), monounsaturated (MUFA), polyunsaturated (PUFA) fatty acids and the highly unsaturated PUFA docosahexaenoic acid (DHA) in different tissues from a range of species including ectotherms, birds and mammals. In addition, we list the species-specific body mass as well as maximum lifespan (MLSP), both obtained from the AnAge database [51]. Whilst levels of mitochondrial SFA, MUFA and PUFA appear to be constant in species with different lifespans, levels of the highly unsaturated lipid DHA, decrease dramatically with increasing lifespan, and this is demonstrated graphically in Figure 4. Using correlative measures only, we find this strong relation for DHA disappears when plotting liver tissue only (not shown) but reappears with plotting heart data (Figure 4D). We acknowledge, however, that the compiled fatty acid profiles from isolated mitochondria in Table 2 and Figure 4 are somehow limited and contain information from very distinct taxa and with large within-rodent taxon clustering. For this reason, we have not conducted statistical analyses with corrections for body mass and phylogeny as this is likely to produce erroneous results. Further work will be required to amass sufficient data from many different species to conduct large-scale analyses.

**Table 2:** Mitochondrial membrane fatty acid composition in a range of tissues from endothermic and exothermic species

**Species (common name)**	**Mass (kg)**	**MLSP (%)**	**SFA (%)**	**MUFA (%)**	**PUFA (%)**	**DHA (%)**	**Tissue**	**Reference**
*Pogona vitticeps*	0.5	12	21.8	29.1	49.1	0.7	Liver	[52]
(bearded dragon lizard)			25.7	26.9	47.4	0.9	Kidney	
			18.6	27.9	53.5	3.3	SKM	
*Bufo marinus*	0.1	24.8	26.3	24.7	49	2.2	Liver	[52]
(cane toad)			19	25.5	55.5	1.4	Kidney	
			17.6	21.9	60.5	2.2	SKM	
*Columba livia*	0.36	35	38.5	13.2	48.3	2.1	Liver	[7]
(pigeon)			43.4	7.8	48.9	1.3	SKM	
			41.1	13	45.9	1.7	Heart	
*Coturnix chinensis*	0.05	5	40.8	15.7	43.5	6.5	Liver	[7]
(king quail)								
*Coturnix japonica*	0.12	6	41.9	14.6	43.6	7.3	Liver	[7]
(Japanese quail)								
*Agapornis* spp.	0.05	16	38.4	19.2	42.5	3.0	Liver	[7]
(lovebird)								
*Nymphicus hollandicus*	0.09	35	42	17.5	40.5	3.8	Liver	[7]
(cockatiel)								
*Eutamias amoenus*			35.6	24.1	40.3	6.47	Liver	[53]
(yellow pine chipmunk)			37.4	12.5	50.1	27.3	Heart	
*Rattus norvegicus*						7.8	Liver	[8,9,52]
						2.1	Kidney	
(rat)	0.35	2.8				14.9	SKM	
			32.9	13	54.2	7.8	Heart	
*Mus musculus*, C57Bl/6	0.02	4	34	11	55	21.9	Liver	**
(house mouse)								
*Mus musculus*, Ames dwarf	0.01	5	38.9	8.8	52.4	9.97	Liver	[54]
mouse (house mouse)								
*Peromyscus maniculatus*	0.02	8.3	32.6	9.75	57.7	34.9	Heart	[55]
(deer mouse)								

DHA docosahexaenoic acid; MLSP, maximum lifespan; MUFA, monounsaturated fatty acid; PUFA, polyunsaturated fatty acid; SFA, saturated fatty acid; **, TGV, unpublished data. SKM skeletal muscle.

**Figure 5:**
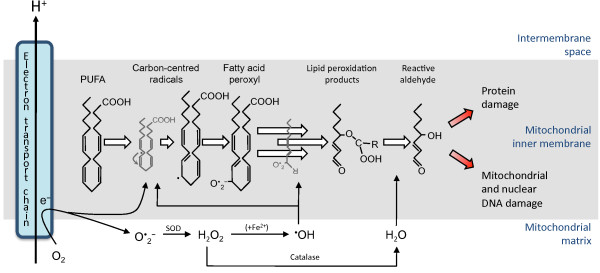
**Mitochondrially generated reactive oxygen species such as superoxide (O**_**2**_^**—**^**) and hydroxyl radicals (•OH) are free to attack methylene bridges adjacent to unsaturated carbon bonds in fatty acids.** This results in the formation of lipid peroxidation products. Subsequent cyclization and beta-scission and can result in the formation of reactive alkenals such as 4-hydroxy-2-nonenal and malonaldehyde. These extremely reactive but more stable species can diffuse from their site of origin and result in protein and DNA damage. PUFA, polyunsaturated fatty acid. SOD Superoxide dismutase.

The mechanism linking mitochondrial membrane unsaturation and aging might be as follows. Mitochondrial free radicals generated as a product of the respiratory chain during oxidative phosphorylation initiate the formation of a lipid radical. The presence of a methylene bridge adjacent to a carbon double bond is particularly susceptible to attack by oxygen free radicals and can form carbon-centred radicals with subsequent propagation of peroxyl radicals. These lipid peroxidation products result in membrane degeneration as well as protein and genome toxicity [56], culminating in aging and death (Figure 5).

Such observations [27,49,50], led Pamplona and Barja to propose the homeoviscous-longevity adaptation hypothesis: namely, that the lower degree of fatty acid unsaturation in longevous animals decreases their sensitivity to lipid peroxidation and macromolecular damage. They have since published two indices, which they suggest causatively correlate with lifespan: the double bond index [13], later refined to the peroxidation index (PI) [57]. Both indices describe the risk of oxidation of unsaturated fats, the explanation again being that lower PI leads to less lipid and downstream protein and DNA oxidative damage and therefore leads to lifespan extension. Valencak has recently independently found that for isolated mitochondria of long-lived Ames dwarf mice, their PI was 13% lower than that of their heterozygous short-lived siblings [54].

However, we wish to point out several caveats with using PI to explain aging and longevity causally. Firstly, PI does not take into account that saturated fatty acids can under certain conditions be more susceptible to oxidation than unsaturated fatty acids, especially some shorter chained fats [58]. Secondly, PI largely reflects the content of one very highly unsaturated PUFA, DHA. Indeed, DHA is really the predominant PUFA that has been linked with MLSP [14,27,49] irrespective of total PUFA levels, which appear not to vary with lifespan [14] (Figure 4). Thirdly, molecules other than lipid peroxides may ultimately be more important in mediating cellular oxidative damage. For example, research in humans and animal models reveals that the aldehydic lipid peroxidation products, 4-hydroxyl-2-nonenal and malonaldehyde (MDA), are more stable than lipid peroxyl radicals and are therefore able to diffuse from their origin to induce greater cellular damage [59].

Interestingly, Montgomery and colleagues recently reported no difference in the fatty acid composition (including n-3 fatty acids) or PI between the long-lived cockatiel (MLSP 35 years) and short-lived quails (MLSP 5.5 years) [7]. Although the animals studied were all aged one regardless of lifespan difference, this suggests at the very least that either membrane composition bears no relation to lifespan, or that membrane composition at a relatively early age does not predict longevity.

An alternative suggestion to the homeoviscous-longevity adaptation hypothesis might be that if lipid peroxides can act as ROS scavengers [60] rather than simply propagators of ROS as traditionally thought, then possibly the higher PUFA levels in shorter-lived animals may simply reflect a physiological adaptation to a stressful milieu.

#### Oxidative stress in the naked mole rat

Buffenstein and colleagues, approached the question as to whether damage generation underlies species longevity by comparing oxidative damage in a long-lived rodent, the naked mole rat (MLSP >28 years) with the comparably sized mouse (MLSP 3.5 years) [61-63]. Surprisingly and contradicting the oxidative stress hypothesis of aging, concentrations of markers of DNA damage and lipid peroxidation were greater in naked mole rats than in mice, even at a young age [62]. This is in line with data that show greater hydrogen peroxide production than expected from naked mole rat mitochondria [64]. Furthermore, contrary to predictions that oxidative stress increases with aging within species, lipid damage levels did not change with age in naked mole rats [62].

Interestingly, naked mole rats do have a membrane composition that fits with the aforementioned theoretical predictions about lifespan [65]. Compared with mice, naked mole rats have one-ninth the content of highly unsaturated DHA, despite maintaining the same overall phospholipid content [65]. Mitchell and colleagues [65] postulate that this lowers their susceptibility to peroxidative damage and state that the original findings for higher levels of lipid peroxides were because the urinary isoprostanes and liver malonaldehyde (MDA) measured in the Andziak study [62] were specific products of arachidonic acid (C20:4n-6) but not of the more unsaturated DHA (C22:6n-3) [62,65]. Furthermore, Mitchell *et al.* found increased plasmenyl lipid levels for the longer-lived naked mole rats compared to mice [65], and postulated, based on previous studies, that they may act as membrane antioxidants [66,67], so explaining the longer lifespan in these species.

However, whilst we would agree that urinary isoprostanes are products of esterified arachidonic acid, MDA is a known product of both arachidonic acid and DHA [59] and reasonably reflects lipid damage in naked mole rats. Additionally, Mitchell and colleagues do not attempt to explain why, if reduced DHA and increased levels of plasmenyl lipids in mole rats provide a protective mechanism against oxidative damage, these animals have increased mitochondrial and nuclear DNA damage as well as increased MDA levels.

Interestingly, the Mitchell study used assumptions based on previous work, which showed that only four fatty acid species are *de novo* synthesized whilst the rest are remodelled by enzymatic deacylation-reacylation [68]. They demonstrated that for naked mole rats compared to mice, the relative balance of fatty acids is shifted away from *de novo* synthesis and towards remodelling [65]. Supposing the assumptions apply correctly, this may reflect a system compensation for high oxidative stress levels, just as Andziak’s work has demonstrated that peroxiredoxin (an important antioxidant) in naked mole rats may suffer high levels of damage in keeping with its specific function [61]. Similarly, increased levels of plasmenyl lipids [65] may be a compensatory mechanism for high oxidative stress rather than a causative link with longevity. Correlations between levels of plasmenyl lipids and lifespan have not been investigated elsewhere and it would be interesting to conduct this work for a broader range of species.

#### Unsaturation in hibernators

The extent to which dietary PUFAs influence mitochondrial membrane phospholipids was first addressed for deer mice [55], chipmunks [53] and golden-mantled ground squirrels [69]. These studies were designed to identify the role of dietary PUFAs on torpor patterns and hibernation, and revealed that dietary PUFAs (for example, supplementary C18:2n-6 or C18:3n-3) led to a 7% increase in mitochondrial PUFA content and that these changes were paralleled by a 2.5°C decrease in minimum body temperature and longer torpor bouts [70,71]. The duration and extent of hypothermic phases were improved by PUFAs through establishing and maintaining high membrane fluidity [72] and lowering enzyme activity, for example, for cytochrome c oxidase [73]. In other words, increased levels of PUFAs allowed for slowed metabolism and reportedly, had very beneficial effects on the survival of the animals [74].

At the time, however, scientists largely overlooked the predictions from the membrane pacemaker hypothesis of metabolism and aging and left unnoticed the fact that membrane unsaturation or PUFA contents in membranes of different tissues consistently increase in all species observed when an animal becomes torpid and lowers its metabolism (cf the membrane pacemaker hypothesis of metabolism). It took two more decades before Gerson *et al.*[75] compared mitochondrial metabolism between torpid and euthermic 13-lined ground squirrels. They observed that during hibernation, respiration and proton leak were suppressed as expected [75]. Unexpectedly given the lower respiration, membrane unsaturation increased while the animal was torpid and lipid peroxidative damage increased twofold as assessed by MDA levels in isolated liver mitochondria [75]. Although in a subsequent study by the same group and using the same species, mitochondrial ROS production appeared to decrease during hibernation, the assay used in that study detected hydrogen peroxide in the cytosol rather than other free radicals produced intra-mitochondrially [76]. Thus, while the pattern of increasing membrane unsaturation in the course of hibernation is consistent [77], lipid peroxidation during hibernation still remains a matter of debate.

#### Cardiolipin: the mitochondrial phospholipid

Unlike other membrane lipids, CL is a dimerically cross-linked phospholipid that, in eukaryotes, is found almost exclusively in mitochondria and almost entirely in their inner membrane [22] (Table 1). This makes it interesting to investigate in terms of the link between mitochondrial membranes and longevity.

Because of its unique dimeric structure, CL has two glycerol backbones each with a chiral centre and four fatty acid chains, making the potential for complexity rather large (Figure 2). In eukaryotic tissues ranging from fungi to mammals, CLs contain mainly monounsaturated or di-unsaturated chains with 16 or 18 carbon atoms. This restricted fatty acid chain length and saturation result in a relatively homogeneous distribution of double bonds and carbon numbers among the four acyl chains [78].

In the mitochondrial inner membrane, CL is involved in stabilising membrane proteins including respiratory complexes [79] and the adenine nucleotide transferase [80]. Furthermore, studies show that CL directly influences the function of the adenine nucleotide transferase [81], an important mitochondrial enzyme that allows the import of ADP into mitochondria for ATP synthesis, and ejects synthesized ATP for use in intracellular processes. In the mitochondrial outer membrane, CL has been suggested to be present in and to be implicated in the function of the protein import machinery of mitochondria (reviewed in [82]). It has also been shown to have a role in regulating apoptosis through several mechanisms including interaction with caspase 8 [83] and cytochrome c [84], as well as playing a vital role in mitochondrial network morphology through interaction with fission/fusion proteins in the outer membrane (reviewed in [82]).

Despite CL’s physiological importance and its partial susceptibility to oxidative damage due to the presence of four unsaturated fatty acid chains, there is weak evidence that CL itself impairs or promotes longevity.

Many studies have used methodological approaches that provide mechanistic insights and possibly allow the authors to comment on CL’s putative role in ‘aging’ but not in lifespan [85]. For example, the response of young and aged mitochondria to exogenously supplemented CL cannot address the role of CL in lifespan [86].

At best, one yeast study showed that impaired CL synthesis lead to decreased longevity, which was restored by enhancing the stress response pathways and promoting cellular integrity using an osmotic stabiliser [87]. Although certain studies showed decreased CL levels in aged worms [88], this was consistent with their finding of decreased mitochondrial numbers and hence membranes. Interestingly, for aged rats, there is some evidence that CL fatty acid chains are remodelled from linoleic acid (18:2n-6) to the more unsaturated arachidonic (20:4n-6) and docosahexaenoic (22:6n-3) acids [89]. There is evidence elsewhere that remodelling occurs in other phospholipid species. In pulse-label experiments of phosphatidylcholine and phosphatidylethanolamine, Schmid *et al.* showed that only four fatty acid species were *de novo* synthesized (6:0–18:2 (n-6), 16:0–18:1, 16:0–22:6 (n-3) and 18:1–18:2 (n-6)), whilst the remainder were remodelled via rapid deacylation-reacylation [68]. This may explain why in a recent phylogenomic study by Jobson [90] examining codon evolution across 25 mammalian species with different longevities, of genes with significantly high evolutionary selection in long-lived species there were a number of lipid membrane composition genes. These were fatty acid elongases, desaturases and fatty acid synthases including those involved in the reconstruction of membrane CLs [90]. Again, these studies may echo our previous suggestion than PUFA levels are a response to cellular stress rather than being a causative agent in aging.

### Phylogenetic and allometric corrections: a beautiful theory killed by an ugly fact?

Simple correlations between the phospholipid composition of mitochondrial membranes, metabolism and longevity bear two notable risks. Firstly, fatty acid composition, as probably all other physiological traits, correlates with body mass because body mass represents a most ‘pervasive trait influencing all aspects of organismal biology’ [91]. By simply relating the DHA content in a given membrane or tissue to maximum lifespan, one might end up having a close correlation between the two but the traits might actually have no functional relation to each other. For example, DHA might be simply more enriched in mitochondrial membranes of a mouse compared to an elephant due to allometry [15]. Secondly, although independent replicates are prerequisites for applying powerful parametric statistical tests, both Speakman [16,91] and Valencak and Ruf [15] point out that different species do not represent independent replicates as they may be phylogenetically correlated despite not sharing the same ancestor.

To overcome both of the above issues, statistical ‘remedies’ that simultaneously correct for body weight and phylogeny have been developed and are freely available online in the form of multivariate regression analysis and the package APE in R [92]. Additionally, this area of research has greatly benefitted from advances in genetics and DNA sequencing, so permitting the accrual of more accurate phylogenetic relationships among species.

Previously, Valencak has found that applying this corrective statistical approach to a large dataset of mammals (using the package Phylogr in R), several of the reported relations fell apart for example, between DHA and MLSP [15]. Similar corrections linking other traits, such as ROS production, with lifespan in a comparative dataset have seen a similar loss of statistical significance once body mass and phylogenetic corrections were made [64]. Therefore, as well as suggesting the re-examination of previous work conducted without accounting for body weight or phylogeny, we recommend that future comparative studies should employ the suggested methodology to allow for better correlation of physiological traits with longevity.

It has been argued by critics of this approach that correcting for body weight and phylogeny might be overly conservative, as it may wipe away important variation in the data that co-explains the observed relations. However, from an evolutionary and comparative physiological perspective, especially in light of improved statistical approaches compared with the older residual analysis approach [93], we suggest the use of body weight- and phylogeny-corrected statistical approaches for all comparative datasets, especially those relating to aging and lifespan. While statistically eliminating the influence of body size might mainly affect the interpretation of comparative datasets, we even previously suggested that, given a large sample with little within-taxon clustering, the incorporation of phylogeny into the models may not affect the interpretation of the main findings but instead give results at a much finer resolution [15]. Undoubtedly, the correction for body weight with the resulting lack of a relation clearly indicates that many of the so-far reported correlations might in fact have been ‘spurious’ [91] or, at least, the magnitude of the association might be much smaller than suggested by simple cross-species correlations.

### Caloric restriction and fatty acid metabolism: all about the omega?

Valencak and Ruf’s use of statistical models that adjust for body weight and phylogeny showed that in contrast to previous studies, there was no relation between MLSP and membrane unsaturation, DHA content or peroxidation index [15]. Only one parameter correlated significantly with lifespan: the ratio of n-3:n-6 PUFAs, with decreased with increasing lifespan. These findings mimic the well-known difference in the n-3:n-6 PUFA ratio between mammals and birds of similar size: the relatively longevous birds have lower n-3:n-6 PUFA ratios [12].

Interestingly, caloric restriction without malnutrition – the only effective physiological means of extending lifespan for a large range of species [94] – results in a decrease in the percentage of n-3 and an increase in n-6 PUFAs [95]. By using Weindruch’s paradigm and calorically restricting mice at different levels, Faulks *et al.* noted a clear decrease in n-3 PUFAs and in the n-3:n-6 ratio in mitochondrial phospholipids from skeletal muscle, liver, brain and heart tissues [96]. Of note, they did not find pronounced differences in ROS production in the animals [96]. However, a more recent study by Valencak shows that despite changes in n3:n6 PUFA ratios (and PI) in mice fed different diets, there was no difference in longevity [26]. From a physiological viewpoint, the significant relation between the n-3:n-6 PUFA ratios and MLSP might be linked to some other feature rather than being causative for senescence and aging.

The literature suggests that the general biochemical and physiological observations for the link between membrane composition and lifespan appear to be supported by genomic studies. This includes a phylogenomic study by Jobson *et al.* showing increased evolutionary selective pressure for genes encoding membrane composition in longer-lived mammals [90] and gene ablation studies of membrane composition genes [97].

However, closer inspection raises uncertainties. The Jobson study [90] does not reflect that higher animals are unable to *de novo* synthesize n-3 and n-6 PUFAs, thus explaining why they might exert a stronger evolutionary selection on elongase and desaturase enzymes. To illustrate this point another way, their work showed that genes ensuring genome integrity did not have a strong selective pressure in longevous animals compared to shorter-lived ones. This is unsurprising given that all animals probably need a similar level of strong control over their genome to prevent cellular and organismal death; but this is not evidence that genome integrity is not important in longevity. Certainly their findings are novel, interesting and require further investigation, including correcting for body mass and investigating species across different taxonomic orders, which are likely to have different selective pressures.

Other studies investigating the effect of ablation of membrane composition pathway genes on lifespan extension are likely to be of huge importance in shedding mechanistic light on the topic; however, contemporary studies suffer from using the same oxidative stress hypotheses to explain lifespan extension without actually measuring oxidative damage species [97].

So while it appears that there is reasonable circumstantial evidence for a link between membrane composition and longevity, there is no evidence of causation. More mechanistic work and a range of species will be needed to decipher how and why membrane composition might correlate with lifespan and whether it is causative.

We suggest that for future studies, the following points ought to be addressed:

● The use of too small a comparative sample, that is, too few species from overly close taxonomic groups. Optimal comparative datasets should contain samples from whichever and as many species as can be sampled.

● The use of potentially inappropriate measures, for example, basal metabolic rate (which represents the minimum energy requirement for staying alive) as opposed to average daily energy use [16]; or using maximum lifespan (clearly an extreme characteristic) rather than the median of an upper percentile of longevous years. Although this issue is contentious, it merits discussion and attention [91].

● Statistical analysis without attention to confounding covariates, for example, fatty acid composition and correlation with lifespan without correction for body mass or phylogeny. Phylogenetic correction is particularly important for comparative studies containing within-taxon clustering where statistical degrees of freedom would be clearly exaggerated if used as independent data replicates.

● Correlation being confused with causation without sufficient evidence or logical premise, or without due attention to confounding mechanisms, for example, polyunsaturated lipid peroxides causing aging rather than being associated with it for some other reason including physiological responses to stress.

## Conclusions

The concept of immortality and longevity has probably captivated humankind from the earliest days and was first recorded by the Babylonians 4,000 years ago. Yet, only in the last two centuries have we made some progress in attempting to answer the question: ‘What makes some organisms live longer?’ Starting from body mass correlations, through to metabolic rate, oxidative stress and membrane fatty acid composition, the field remains complex and burdened with irregularities.

We conclude from reviewing the literature available on mitochondrial lipid composition that there may well be an association between high levels of membrane n-3 PUFAs and a comparatively low longevity, as would be predicted by the homeoviscous-longevity adaptation or the membrane pacemaker hypotheses of aging. However, our overview also suggests that the magnitude of this association might vary according to the tissues and datasets used (such as the specific animal taxa or even knock-out mouse models). It is currently difficult to appreciate whether and which tissues are important in correlations between membrane composition and longevity. Likewise, the extent of the relation between membrane composition and MLSP might have been overestimated in the past due to various reasons, including the lack of powerful statistical approaches built on reliable phylogenetic information. Comparative datasets that do not correct for phylogeny or co-variation of traits with body mass may easily lead to oversimplified relations for certain physiological traits and maximum lifespan. The statistical approaches developed by comparative biologists may be powerful tools for getting new and more accurate information out of comparative differences in the aging process across phylogenetically very different species.

On a side note, we observe a difference in the perception as to which fatty acid classes are beneficial or detrimental to human health span. Among comparative physiologists, saturated fatty acids are considered rather harmless constituents of membranes and tissues. They have a fixed amount in membranes and bring about little if any cellular damage due to peroxidation. In contrast, nutritionists and physicians perceive that saturated fats bring about an increased risk of cardiovascular disease. Abbott and colleagues recently showed that extensive changes in the SFA, MUFA and PUFA levels of diets had minimal effects on the fatty acid composition of membranes in rats but considerable influence on adipose tissue and plasma triglycerides [98]. This likely explains the difference in perception and fits in with epidemiological and clinical evidence suggesting that mortality due to coronary heart disease can be reduced by partly replacing dietary saturated fats with polyunsaturated fats while maintaining a low intake of trans fatty acids [99,100]. This dietary modification would reduce serum concentrations of triglycerides and cholesterol, which is a well-established risk factor for heart disease [101].

## Abbreviations

CL: cardiolipin; DHA: docosahexaenoic acid; FA: fatty acid; MDA: malonaldehyde; MIM: mitochondrial inner membrane; MLSP: maximum lifespan; MOM: mitochondrial outer membrane; MUFA: monounsaturated fatty acid; PI: peroxidation index; PUFA: polyunsaturated fatty acid; ROS: reactive oxygen species; SFA: saturated fatty acid; UCP: uncoupling protein.

## Competing interests

The authors declare that they have no competing interests.
